# The Functions of Phasic Wing-Tip Folding on Flapping-Wing Aerodynamics

**DOI:** 10.3390/biomimetics9030183

**Published:** 2024-03-18

**Authors:** Yiming Li, Keyu Li, Fang Fu, Yao Li, Bing Li

**Affiliations:** 1 Guangdong Provincial Key Laboratory of Intelligent Morphing Mechanisms and Adaptive Robots, Harbin Institute of Technology, Shenzhen 518055, China; 2 Key University Laboratory of Mechanism & Machine Theory and Intelligent Unmanned Systems of Guangdong, Harbin Institute of Technology, Shenzhen 518055, China; 3 School of Mechanical Engineering and Automation, Harbin Institute of Technology, Shenzhen 518055, China; 4 College of Art and Design, Shenzhen University, Shenzhen 518060, China

**Keywords:** flapping-wing flight, wing-tip folding, insect flight aerodynamics, bioinspired flapping mechanism

## Abstract

Insects produce a variety of highly acrobatic maneuvers in flight owing to their ability to achieve various wing-stroke trajectories. Among them, beetles can quickly change their flight velocities and make agile turns. In this work, we report a newly discovered phasic wing-tip-folding phenomenon and its aerodynamic basis in beetles. The wings’ flapping trajectories and aerodynamic forces of the tethered flying beetles were recorded simultaneously via motion capture cameras and a force sensor, respectively. The results verified that phasic active spanwise-folding and deployment (PASFD) can exist during flapping flight. The folding of the wing-tips of beetles significantly decreased aerodynamic forces without any changes in flapping frequency. Specifically, compared with no-folding-and-deployment wings, the lift and forward thrust generated by bilateral-folding-and-deployment wings reduced by 52.2% and 63.0%, respectively. Moreover, unilateral-folding-and-deployment flapping flight was found, which produced a lateral force (8.65 mN). Therefore, a micro-flapping-wing mechanism with PASFD was then designed, fabricated, and tested in a motion capture and force measurement system to validate its phasic folding functions and aerodynamic performance under different operating frequencies. The results successfully demonstrated a significant decrease in flight forces. This work provides valuable insights for the development of flapping-wing micro-air-vehicles with high maneuverability.

## 1. Introduction

The study of micro-air-vehicles has become a highly active field of research in recent years, with significant advancements in the development of these small flying machines. Due to their compact size, micro-air-vehicles have the ability to navigate through narrow spaces, making them highly versatile and efficient. Furthermore, micro-air-vehicles could have promising practical applications in fields such as search and rescue operations [1,2], and environmental monitoring [3,4]. Micro-air-vehicles predominantly employ flapping-wing flight methods, which imitate the flight principle of insects. The flapping-wing flight mode offers superior energy efficiency when the wings are small, a crucial advantage for micro-air-vehicles where energy consumption is a key factor [5].

Researchers have long been fascinated by the idea of creating micro-air-vehicles that could replicate the flight capabilities of insects. In recent years, significant progress has been made in the development of flapping-wing micro-air-vehicles (FWMAVs). RoboBee is a breakthrough work in FWMAVs. Despite its small size and light weight, RoboBee is able to achieve hovering in the air, and flying horizontally through a complex, dynamic, and mechanical design [6]. In subsequent research, researchers also achieved underwater take-off [7] and surface electrostatic adhesion [8], which further expand the application scope of RoboBee. In addition, Chen et al. used a DEA as the actuator and adopted a multi-machine parallel approach to maintain stable flight in complex environments [9]. Fuller et al. designed a small optoelectronic conversion circuit that can power a robot through a high-energy laser beam, freeing the robot from the constraints of a wire harness [10]. Some other studies focused on novel drive and control methods for insect-scale FWMAVs. Hines et al. presented a single motor-driven FWMAV capable of controlling individual wing flapping angles, resulting in a maximum lift-to-weight ratio of 1.4 at 10 Hz for a 2.7 g system [11]. Apart from conventional motor drives, novel electromagnetic actuators [12] and DEAs [13] have both been shown to be feasible to drive miniature flapping-wing robots. However, efficient and flexible flight requires not only a superior drive method but also an efficient drive mechanism and control method. A combination of four-bar linkage and pulley-string mechanisms were employed to develop a flapping mechanism capable of realizing a wide flapping amplitude of ~90°, successfully achieving vertical climbing, hovering, and loitering [14]. A number of insect-inspired passive wing-folding mechanisms and control methods have also proliferated, as detailed in [15,16,17,18,19].

Despite remarkable advancements in the study of FWMAVs in recent years, even state-of-the-art prototypes still lag behind insects in terms of maneuverability and flexibility. This gap highlights the need for continued research and improvement in the field to bridge the gap between artificial and natural flight capabilities. One of the main reasons for this disparity lies in inadequate understanding of insect flight mechanics. For instance, the precise dynamic models by which insects control their flight orientation by changing their wing and body kinematics remain an active area of research [20]. Additionally, FWMAVs are constrained by their size and payload, which limits their attitude adjustment capabilities and renders their flight trajectories susceptible to instability. To address these challenges, it is essential to strengthen our research on insect flight mechanisms. With a deeper understanding of natural flight phenomena, we can apply these valuable insights to the design of FWMAVs.

Insects achieve efficient flight through rapid and powerful wing beats. Their remarkable ability to flexibly adjust wing-beat frequency and stroke trajectory allows them to seamlessly adapt to diverse flight environments, effortlessly executing swift take-offs, stable hovering, and sharp turns. For instance, forward or backward body pitching can be induced by an upward or downward shift of the line of action of the stroke trajectory [21,22,23]. Even in adverse conditions such as wind or unpredictable airflow, insects can promptly restore flight stability through subtle adjustments to wing angle [24]. With exceptional maneuverability, insects can easily escape from predators, and migrate for long distances. Furthermore, the structural strength and resilience of insect wings enable them to endure various stresses and deformations throughout flights.

In this study, we investigated the flapping flight process of beetles and uncovered a novel mechanism for modulating flight forces. On rare occasions, beetles were observed to fold their wing-tips in certain phases during flapping cycles, dramatically altering their flight forces without any changes in flapping frequency. This novel flapping flight control presents ideas for the design of a micro-flapping-wing mechanism. Therefore, we have designed a bioinspired flapping mechanism that mimics the phasic wing-tip folding of beetles, utilizing a cable-driven mechanism. Our innovative design has successfully demonstrated a significant decrease in flight forces, paving the way for future developments in braking and saccade control of FWMAVs.

## 2. Results and Discussion

### 2.1. In-flight Wing-Tip Folding and Its Aerodynamic Effects

**Figure 1:**
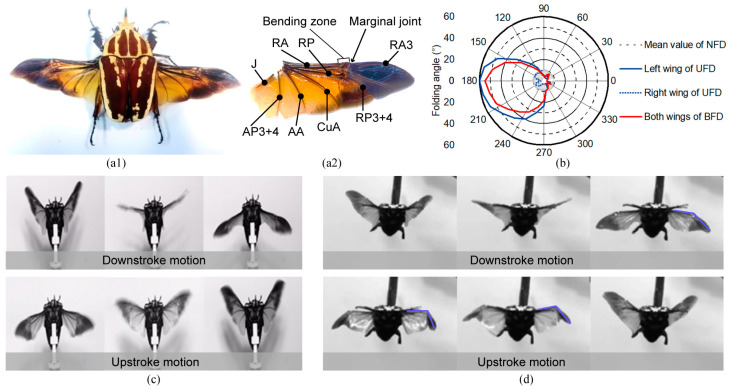
Phasic wing-tip folding in beetles. (**a1**,**a2**) A beetle with wings deployed and the detail of its wing veins. The blue-shaded area denotes the foldable wing-tip area. (**b**) Polar plot of the folding-angle phase of beetles in flapping flight. The zero-phase angle corresponds to the highest position of the wing in a stroke cycle. The folding-angle-variation curves for unilateral folding and deployment (UFD) are shown in blue, and bilateral folding and deployment (BFD) is shown in red. NFD denotes normal flight with no folding and deployment. (**c**) Snapshots captured by high-speed camera illustrate the flapping flight processes of NFD and (**d**) BFD.

The newly discovered wing folding in flying beetles is different from the previous understanding that beetles can only fold their wings passively in flapping flight [17] and that active wing-folding motion is used for flight cessation. To explore the wing-folding process and the resulting effects on beetles’ flight, we performed experiments and observed normal flapping flight with no folding and deployment (NFD) and flapping flight with wing folding in tethered flying beetles (*M. torquata*) [see Figure 1(a1)]. As shown in Figure 1c,d, compared to normal NFD flight, wing folding occurred in the wing-tip area. Specifically, active wing-tip folding of the flying beetle was observed to occur primarily during certain phases of the upstroke motion, with the wing-tip producing a noticeable spanwise bend around the marginal joint [see Figure 1(a2)]. On nearly reaching the end of the upstroke motion, the wing-tip deployed again. Typically, two kinds of wing-tip-folding phenomena were found in *M. torquata* beetles, namely unilateral folding and deployment (UFD) and bilateral folding and deployment (BFD).

**Figure 2:**
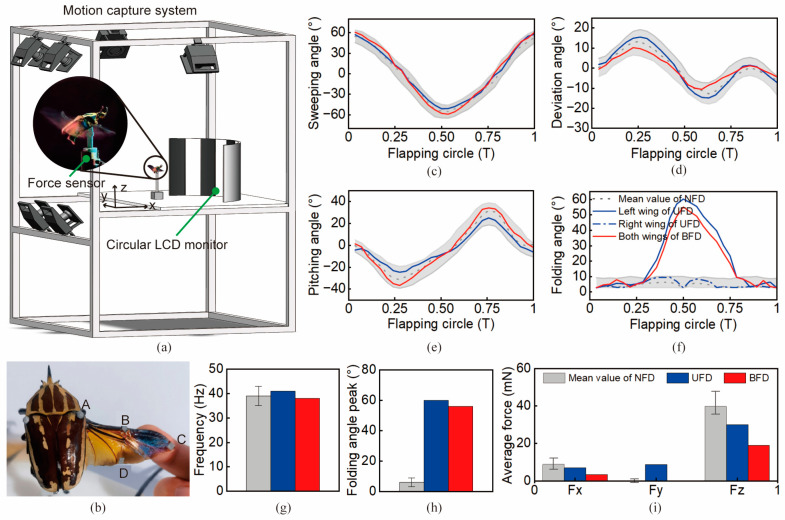
Wing-stroke measurements of tethered flying beetles. (**a**) The motion capture system was used to track and reconstruct wing-stroke motion trajectories. A force sensor was adopted to measure the aerodynamic forces. (**b**) Eight reflective markers were attached on the flying beetles (four markers per side). The markers can be tracked by the motion capture cameras to reconstruct the wing trajectories. (**c**–**f**) Sweeping-angle, deviation-angle, pitching-angle, and folding-angle-variation curves during each flapping circle. The areas shaded in gray denote the normal angle range of NFD flight with extreme value characterization. (**g**) Flapping frequencies of NFD, UFD, and BFD. (**h**) Folding-angle peaks of flights with NFD, UFD, and BFD. (**i**) Average aerodynamic forces of NFD, UFD, and BFD. Fx, Fy, and Fz denote forward thrust, lateral thrust, and longitudinal lift, respectively.

To investigate the effect of wing-tip folding on the wing-stroke motion of flying beetles, we first investigated the flapping angles and folding angles of the beetles during normal flight (*N* = 10 beetles and *n* = 50 trials). The variation ranges of flapping angles and folding angles in NFD flapping of the beetles were obtained via tethered flight experiments. From the flapping angle curves in a cycle [see Figure 2c–e], it can be seen that the mean values of the amplitudes of sweeping, deviation, and pitching angles were 107.6°, 29.1°, and 62.6°, respectively. These values provide a parametric reference and comparison metric for subsequent analyses and micro-flapping-wing mechanism design.

Wing-stroke observations of tethered flying beetles demonstrated that wing-tip folding occurred periodically at specific phases. As shown in Figure 1b and Figure 2f, two wing-tip-folding phenomena, UFD and BFD, always appeared at the same phase angles. The folding angles of both UFD and BFD started changing significantly at the phase angle of ~110° (~1/3 T), increasing beyond the normal range. Until the phase angle reached 180°, the folding angle reached its maximum value of ~60°. Similarly, the folding angles of both UFD and BFD deployed and returned to the normal range at a phase angle of ~290° (~5/6 T). The wing-folding phenomenon is well tuned with wing-stroke cycle. However, bilateral asymmetrical folding of the wings in UFD revealed that folding control is independent from flapping control. Thus, phasic active spanwise-folding and deployment (PASFD) is found in beetle flight control. This newly discovered PASFD phenomenon further confirms and complements Hass’s conjecture about the possible existence of phasic active claval-wing-folding in beetles (which was not experimentally confirmed due to the low frame rate of digital cameras at that time) [25].

When comparing the flapping motion with and without folding, we found that both the flapping frequencies were essentially stable and constant. As shown in Figure 2g, the flapping frequencies of UFD and BFD were 41 Hz and 38 Hz, respectively, neither of which was significantly different from that of normal flapping flight (39.3 ± 3.7 Hz), demonstrating the inability of the beetles to actively adjust their flapping frequencies when folding wings. In fact, the flapping motions of beetles are dominated by their asynchronous flight muscles, whose frequency is decided by the thorax structure rather than neural inputs [26]. As shown in Figure 2h, the peak folding angles for both UFD and BFD were ~60°, while the folding angle for normal flight without folding was substantially negligible (~6 ± 3°). Moreover, the folding angles in UFD and BFD flights remained the same, implying that the folding angles are used in flight control as a simple on–off output.

The PASFD of flying beetles considerably affects aerodynamics. As shown in Figure 2i, compared to normal NFD flight, the forward thrust and longitudinal lift of BFD were significantly reduced. Specifically, flight lift was reduced by 52.2% and forward thrust by 63.0%, but lateral thrust did not obviously change. However, for UFD, lift and forward thrust reduction was not as much (~21.7% and ~24.6%), but this unilateral folding induced a noticeable lateral thrust decrease (8.65 mN). Thus, the use of BFD can be an effective way to decrease flight aerodynamic forces rapidly, which provides another new idea for deceleration control in beetles’ free flight [27]. Moreover, UFD control is an advanced flight control method that is beneficial to increase the lateral maneuverability and agility of flapping flyers, which is only found and verified on birds [28].

### 2.2. Bionic Design of the Flapping Mechanism

**Figure 3:**
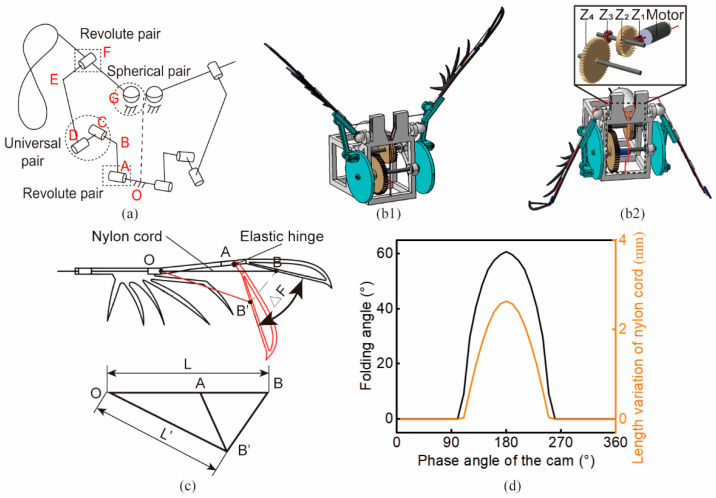
Design of the beetle-inspired micro-flapping-wing mechanism. (**a**) The RURS mechanism proposed in [29] was used as the flapping-wing driving mechanism. It is comprised of two revolute pairs, a universal pair, and a spherical pair. (**b1**,**b2**) Three-dimensional CAD model of the micro-flapping-wing mechanism and its detailed secondary gear reduction mechanism. The former demonstrates the wings fully deployed at the highest position in the flapping cycle, and the latter illustrates the wings folded into the largest angle at the lowest position. (**c**) The cable-driven method was chosen to drive the elastic hinge. High-tenacity nylon cord was used for the folding of the elastic hinge. The three points O, A, and B were simplified to be on a straight line, and then as the pulled wing-tip rotates around the hinge point A (from AB to AB′), the length variation of the nylon cord can be calculated by ∆L = L − L′. (**d**) Relationship between the phase angle of the cam, length of the nylon cord, and folding angle of the wing.

To mimic the PASFD of flying beetles, we designed a beetle-inspired flapping mechanism, focusing on the function of flapping-swing-twist motion. To generate the three-dimensional (3-D) trajectory of the flapping-swing-twist motion of a beetle in flight, the Revolute-Universal-Revolute-Spherical (RURS) mechanism [29] was chosen as the base configuration for the stroke motion. The RURS mechanism consists of three types of moving pairs: revolute, universal, and spherical pairs. Afterwards, a multi-objective optimization method was employed to solve the optimal connecting rod length using the flapping angle amplitude results from Figure 2c–e. The revolute pair of the final RURS-based flapping mechanism was driven by a single motor, and its output end with the specially designed connecting rod length can realize the “8”-shape 3-D trajectory [see Figure 3a]. Furthermore, we specially designed the cable-driven folding mechanism, using nitrile rubber as the elastic hinge. The elastic hinge mimics the function of resilin in biological wings, and is capable of storing elastic energy to provide the energy needed to unfold the wing [25]. Specifically, tightening of the nylon cord (from OB to OB′, see Figure 3c) results in elastic energy storage, and when the cord is loosened (from OB′ to OB) the elastic energy drives the deployment of the wing-tip.

**Figure 4:**
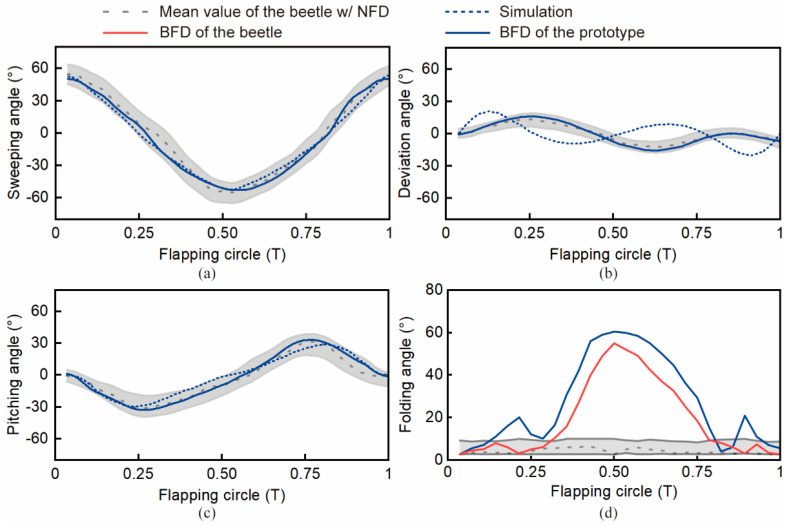
Simulation and testing results of flapping angles and the folding angle of the designed micro-flapping-wing mechanism. (**a**–**c**) Sweeping angle, deviation angle, pitching angle variation curves during one flapping circle. (**d**) Folding angle variation curves during one flapping circle. Simulation results of the sweeping angle, deviation angle, and pitching angle were obtained via ADAMS software.

The ADAMS kinematic simulation results demonstrated the flapping-angle variation of the flapping mechanism during one flapping cycle, as shown by the blue dashed lines in Figure 4a–c. From Figure 4a, it can be seen that the variation range of the sweeping angle in one flapping cycle was -55°~55° (i.e., the amplitude was 110°), which was fully consistent with the experimental results (110°). As shown in Figure 4c, the variation range of the pitching angle was −30°~30° (i.e., the amplitude was 60°), which was only 8% different from the experimental value (65°). Figure 4b shows the variation range of the deviation angle was −22.5°~22.5°, which was slightly different from the experimental observation (35°). The simulation result errors of the three angles with respect to the theoretical values are related to the different values of the weighting coefficients (w1 = 0.6 for the sweeping angle, w2 = 0.1 for the deviation angle, and w3 = 0.3 for the pitching angle). In summary, the sweeping and pitching angles, which had a greater effect on lift, revealed small and negligible errors, and the magnitudes were within the measured range of beetle flight, indicating that the mechanism is analogous to flying beetles.

### 2.3. Testing of Wing Flapping and Folding Angles and Measurement of Aerodynamic Forces

The micro-flapping-wing mechanism prototype, consisting of the RURS mechanism, cable-driven folding mechanism, gear reduction mechanism, and supplementary components [see Section 3 Materials and Methods section], was tested with five trials under the motion capture system to validate its performance. Figure 4a–c illustrates that the sweeping angle, deviation angle, and pitching angle of the flapping mechanism prototype were within the normal flapping angle range of the flying beetles. Further, compared to the ADAMS simulation results, the tested sweeping angle amplitude of the prototype was 105°, with an error of 4% from the simulation results; the tested deviation angle amplitude of the prototype was 35°, with an error of 22% from the simulation results; the tested pitching angle amplitude of the prototype was 65°, with an error of 7% from the simulated results. In general, from the preceding results, we can see that the three tested flapping angles of the prototype fit well with both the simulation results and the normal range of the flying beetles.

The variation range of the folding angle of the prototype was also well fitted to that of the tethered flying beetles. As shown in Figure 4d, a slight difference (~5°) was found between the folding angle of the beetles with BFD and that of the prototype. Simultaneously, a surging local peak angle of ~20° existed at the both sides of the prototype folding angle curve. To evaluate the folding angle differences between the prototype and the flying beetles, the root mean square errors (RMSEs) were calculated. The RMSE of the folding angle of the prototype and the beetles was ~2.97°, which was due to the inertia of the elastic hinge during the transition between the upstroke and downstroke motion of the wing, which led to a diagonal upward or downward movement of the wing, resulting in an increase in the folding angle.

**Figure 5:**
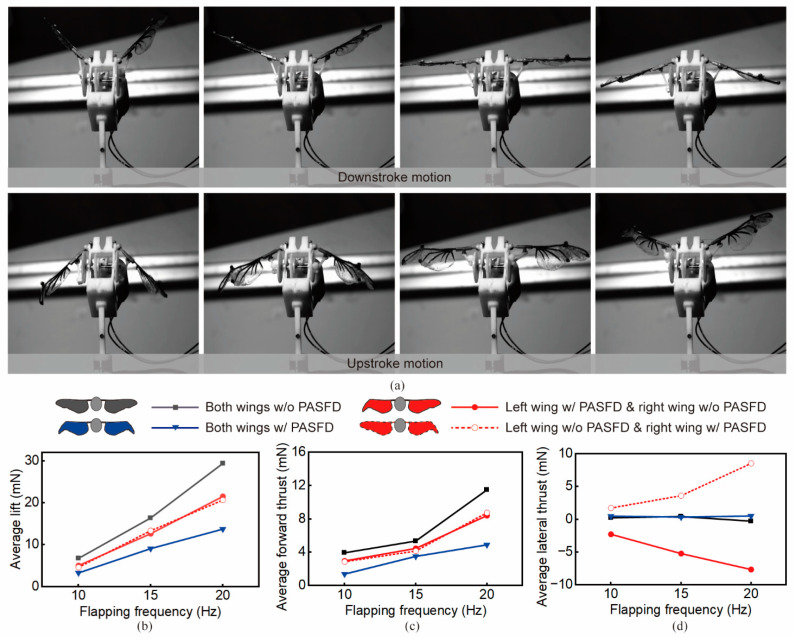
Aerodynamic forces test of the beetle-inspired micro-flapping-wing mechanism. (**a**) Snapshots captured by high-speed camera illustrate the PASFD function of the prototype. The flapping-wing mechanism folds and deploys its wing-tips in certain phases during flapping circles. (**b**–**d**) Average lift, forward thrust, and lateral thrust under different flapping frequencies.

Aerodynamic force is an important metric to characterize the aerodynamic performance of the flapping-wing mechanism. Herein, we tested the aerodynamic forces of the developed prototype under four operating conditions (both wings with and without PASFD, left wing with PASFD, and right wing with PASFD, as shown in Figure 5), using three flapping frequencies (10 Hz, 15 Hz, and 20 Hz) for each set of conditions. Figure 5a shows the wingbeat sequence in one flapping cycle at a flapping frequency of 20 Hz, demonstrating the function of the prototype to successfully fold and deploy the wing-tips during the upstroke motion.

The developed beetle-inspired micro-flapping-wing mechanism prototype exhibited aerodynamic performance similar to that of flying beetles. As shown in Figure 5b–d, at the same flapping frequency, the average lift and forward thrust of BFD (*n* = 5 trials for each flapping frequency) were lower than those of the NFD case. For example, at 20 Hz, the lift and forward thrust of BFD were reduced by 53.4% and 57.3%, respectively. Moreover, NFD and BFD were basically free of lateral thrusts. For both UFD cases (left wing with PASFD or right wing with PASFD), based on their flight forces results, we can notice the presence of significant lateral thrust. Specifically, left-wing folding and right-wing folding generated lateral thrusts of 7.65 mN and 8.52 mN, respectively, at a flapping frequency of 20 Hz. These aerodynamic properties of the PASFD of the wings are consistent with the flight dynamics of flying beetles.

A larger flapping frequency results in a higher flight force. As shown in Figure 5b, the average lift for the four operating conditions basically showed a linear growth relationship when the flapping frequency was from 10 Hz to 20 Hz. However, the average forward thrust became increased nonlinearly in relation to the flapping frequency. As shown in Figure 5c, for NFD and UFD operating conditions, the forward thrusts increased slowly (~1.4 mN increment for both NFD and UFD) when the flapping frequency was increased from 10 Hz to 15 Hz. The faster rise (~6.1 mN increment for NFD and ~4.3 mN increment for UFD) of forward thrusts occurred from 15 Hz to 20 Hz. In contrast, the forward thrust of BFD had a relatively flat trend with increasing frequency (~1.37 mN).

Although artificial FWMAVs have achieved flight balance and hover capabilities comparable to insects, they are still far less maneuverable than insects. Many of the acrobatic flight mechanisms of insects are still not well understood. In this study, we have discovered a new flight control mechanism of beetles, which can achieve rapid deceleration or deflection in flight through simple control. On this basis, a flying flapping wing mechanism, which realizes phasic wing-tip folding, was proposed. The wing-tip folding of the mechanism successfully caused a rapid reduction of aerodynamic forces, offering potential for applications such as flight braking or saccade flight. Unlike bees [30,31] and moths [32], flying beetles are unable to recover from aerial perturbation by modulating their wingbeat frequency. The aerodynamic adjustments in flying beetles appear to be dependent only on wing and wing-tip trajectories [33,34] due to the fact that their flapping frequency cannot typically be actively altered. Thus, it is possible for the beetle-inspired micro-flapping-wing mechanism to achieve better mobility than its natural exemplar through combining phasic wing-tip folding control with flapping frequency control to adapt to different aerodynamic requirements.

## 3. Materials and Methods

### 3.1. Study Animals

The beetle *M. torquata* was selected for this study. Males aged within three months had the best flight ability, so males ranging from 1–2.5 months were adopted (body length: 60.5 mm ± 2.5 mm; unilateral wingspan: 61.5 mm ± 3.5 mm; mass: 6.5 g ± 0.5 g). These beetles were fed with sufficient beetle jelly every three days. Feeding temperature was maintained at ~23 °C with a relative humidity of ~60%. Strict feeding conditions ensured that each beetle used for the experiment had sufficient flight vigor.

### 3.2. Flapping Angles and Folding-Angle Acquisition

The flying beetle was attached to a 3-D-printed bracket at its sternum, which was placed ~40 cm in front of a circular LCD monitor, as shown in Figure 2a. The body pitch angle of the beetle ranges from 25° to 35° during natural flight [27], so the tilt angle of the bracket was designed to be a tradeoff of 30°. A 3-D motion capture system (6 × Vicon V5 cameras; Oxford Metrics Ltd., Oxford, UK) was used to track the wing-stroke trajectories of beetle-tethered experiments at 1000 frames per second. The motion capture system could accurately capture and track the positions of reflective materials; thus, reflective markers were used to record the wing-stroke motion of the beetle. Specifically, two reflective markers (diameter: 3 mm) were glued on both sides of both wing bases, and six reflective strips (6 mm × 3 mm) were affixed to the wings on both sides, as shown at the A, B, C, and D positions in Figure 2b. The marker coordinates can be calculated and exported directly by the computer software (Vicon Nexus v2.6.1) of the motion capture system.

**Figure 6:**
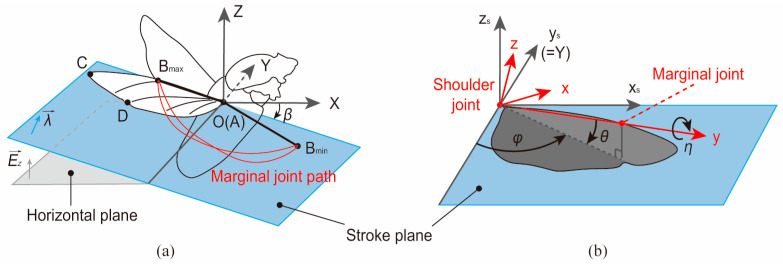
Schematic showing the coordinate systems and wing-stroke motion parameters. (**a**) Definition of the beetle’s stroke plane. (**b**) Definition of the beetle’s flapping angles.

The flapping angles of the beetle’s wings can be calculated from the marker coordinate vectors within the coordinate system established based on the stroke plane. The coordinate systems presented by Sehyeong Oh [35] were used to describe the wing-stroke motion of the beetle. As shown in Figure 6a, the shoulder joint of the beetle’s right wing was defined as the origin of the coordinate system [XYZ], where the XY plane denotes the horizontal plane. The schematic illustration in Figure 6b shows the flapping angles during the wing-stroke motion, where [x_s_y_s_z_s_] denotes the stroke coordinate system. [xyz] signifies the wing coordinate system, where x, y, and z denote the wing’s thickness-wise, span-wise, and chord-wise directions, respectively. The stroke plane angle, deviation angle, sweeping angle, pitching angle, and folding angle of the beetle’s wing were calculated based on the aforementioned coordinate vectors of the four markers A, B, C, and D.

Stroke plane angle β. As the angle between the stroke and horizontal planes, it can be calculated from the normal vectors of the horizontal plane and the stroke plane in the following equation:

(1) $$\left\{ \begin{array}{l} \vec{\lambda }=\vec{B_{\max}A}\times \vec{B_{\min}A}\\ \beta =\arccos(\vec{\lambda },\vec{E_{z}}) \end{array}\right.$$

where $\vec{\lambda }$ and $\vec{E_{z}}$ denote the normal vectors of the stroke plane and the horizontal plane, respectively.

Deviation angle θ. The deviation angle is defined as the angle between the marginal–shoulder joints line and the stroke plane, which can be calculated by the following equation:

(2) $$\theta =90^{\circ }-\arccos(\vec{\lambda },\vec{\mathrm{AB}})$$

Sweeping angle φ. The sweeping angle is defined as the angle at which a straight line between the wing-tip and the shoulder joint sweeps across the stroke plane during stroke motion. This angle can be calculated from the projection of vector $\vec{\mathrm{AB}}$ on the stroke plane with the direction vector of the spanwise direction.

(3) $$\varphi =\arccos(\vec{\mathrm{AB}}\cdot \cos\theta ,\vec{E_{y}})$$

Pitching angle η. The pitching angle is the angle of rotation around the straight line between the wing-tip and the shoulder joint. It can be calculated by the vector $\vec{\mathrm{BD}}$ and $\vec{\lambda }$.

(4) $$\eta =\arccos(\vec{\mathrm{BD}},\vec{\lambda })$$

Folding angle f. The folding angle is defined as the angle at which the wing-tip folds around the hinge point during the flapping process. This angle can be calculated by the following equation:

(5) $$f=\arccos(\vec{\mathrm{AB}},\vec{\mathrm{BC}})$$

### 3.3. Aerodynamic Forces Measurement

As shown in Figure 2a, a precise six-axis force sensor (Nano17, ATI Industrial Automation, Apex, NC, USA) was used to monitor the aerodynamic forces of the beetle’s flight because it generates lift and thrust in the order of millinewtons. A multi-channel acquisition card (NI USB-6210) was used for the recording of forward thrust, lateral thrust, and lift signals for wing-stroke motion. It should be noted that the force sensor was mounted with its XYZ directions corresponding exactly to the forward, lateral, and vertical directions of the tethered beetle, which ensures that the measured force data correspond directly to the aerodynamic forces in the three directions of the beetle’s flight. The recorded digital signal data were transformed into corresponding force data by means of the force sensor’s factory calibration matrix. The data filtered by the median filter algorithm were used as the final experimental results.

### 3.4. Prototype Design and Fabrication

The RURS mechanism [see Figure 3a] was employed to achieve the distinctive “8”-shape trajectory. The length of each connecting rod in this mechanism was solved by a multi-objective optimization method. Based on the sweeping, deviation, and pitching angle amplitudes measured in the beetle wing-stroke motion experiments, the following three objective functions with respect to the Euler angle were set:

(6) $$\begin{array}{l} f_{1}(X)=\min\left|(\varphi_{\max}-\varphi_{\min})-\varphi_{m}\right|\\ f_{2}(X)=\min\left|(\theta_{\max}-\theta_{\min})-\theta_{m}\right|\\ f_{3}(X)=\min\left|(\eta_{\max}-\eta_{\min})-\eta_{m}\right| \end{array}$$

where $\varphi_{m}$, $\theta_{m}$, and $\eta_{m}$ denote the mean values of sweeping, deviation, and pitching angle amplitudes measured by the beetle wing-stroke experiments, respectively.

Three weighting coefficients w1, w2, and w3 were introduced to the comprehensive objective function (Equation (7)) because of the different degrees of aerodynamic influence of sweeping, deviation, and pitching angles during insect flight [36]. Therefore, the multi-objective optimization problem can be transformed into a single-objective optimization problem. The length of each connecting rod in the RURS mechanism can be obtained by solving the single-objective function using a nonlinear programming algorithm and combining it with the Denavit–Hartenberg (D–H) method to establish a coordinate transformation.

(7) $$f(X)=w_{1}f_{1}(X)+w_{2}f_{2}(X)+w_{3}f_{3}(X)$$

The results in the literature [36] demonstrated that the influence degree of the sweeping angle, deviation angle, and pitching angle on aerodynamic forces during insect flight are 60%, 10%, and 30%, respectively; thus, w_1_, w_2_, and w_3_ in Equation (7) were taken to be 0.6, 0.1, and 0.3, respectively. The multi-objective optimization results for the given rod length OA = 2 mm and AB = 22 mm were BC = 4.45 mm, CD = 5.14 mm, OG = 13.45 mm, DE = 21.45 mm, and FG = 11.09 mm. The 3-D CAD model with optimized rod length is shown in Figure 3b. The theoretical flapping angles based on these rod length results calculated by coordinate transformation were: $\varphi_{\mathrm{theoretical}}$ = 110°, $\theta_{\mathrm{theoretical}}$ = 35°, and $\eta_{\mathrm{theoretical}}$ = 65°. These angle amplitudes were all within reasonable ranges for beetle flight.

The drive motor and gear mechanism were also specially selected and designed to meet the power input of the flapping-wing mechanism. A high-speed hollow-cup motor (CHR-R370, rated voltage: 11.1 V, max. speed: 75,000 rad/min) was used as the power source. A secondary gear reduction mechanism (z1 = 8, z2 = 26, z3 = 10, z4 = 44) with a ratio of 14.3 was designed as a torque multiplier for the motor as shown in Figure 3(b2). The gear mechanism transmission efficiency was considered in the design process, after which the output rod of the whole flapping-wing driving mechanism can reach 30 Hz frequency, which was within the common flapping frequency range of beetles.

**Figure 7:**
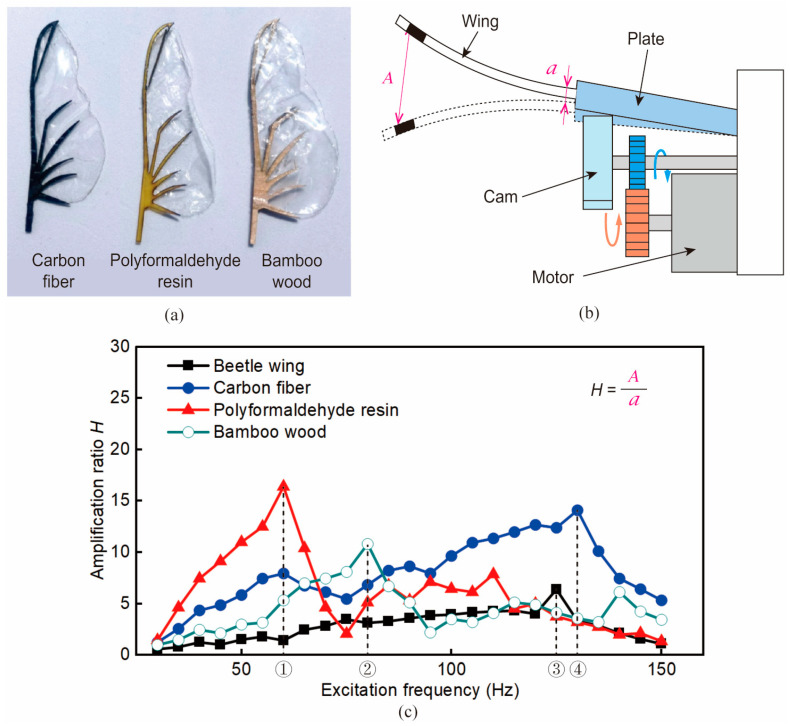
Material selection of the wing vein. (**a**) Three materials were used for wing vein fabrication. The three wing veins were made of carbon fiber, paraformaldehyde resin, and bamboo wood from left to right. The wing membranes attached thereto were made of polyethylene film. (**b**) The intrinsic frequency test rig [37]. (**c**) Results of intrinsic frequency tests of the beetle wings and artificial wings. The positions ①, ②, ③, and ④ indicate the frequencies corresponding to the maximum amplification ratios of different materials, respectively, i.e., the intrinsic frequency of that material.

The vein material of artificial wings has an important influence on flapping performance. Specifically, wing vein materials are not only required to have suitable material strength and density, but also the suitability of the intrinsic frequencies of different materials to that of flying beetles’ wings needs to be taken into account. In terms of wing vein material selection, three commonly used materials, carbon fiber, polyformaldehyde resin, and bamboo wood, were considered as choices for comparison [see Figure 7a]. The properties of these three materials are shown in Table 1.

The intrinsic frequency test method proposed by Naka et al. [37] was employed to measure the intrinsic frequency of wings made of three different materials. The principle of the test rig was that a motor drove a cam to rotate to cause wing vibration. Wing veins made of each of the three materials were attached to the plate and given an excitation frequency of 30 Hz to 150 Hz for vibration testing. It should be noted that the wing membranes of all three types of wings were made of 50 μm thick polyethylene film. The ratio of the displacement *A* at the wing-tip to the displacement *a* near the plate end was defined as the amplification ratio *H*. The amplification ratio *H* was used to evaluate the intrinsic frequencies of the three artificial wings at each excitation frequency.

Figure 7c illustrates the amplification ratio curves for the different wing materials. The excitation frequency corresponding to the position with the largest amplification ratio is the intrinsic frequency of the material [37]; thus, it can be seen that the intrinsic frequency of the polyformaldehyde resin was the lowest (65 Hz); the intrinsic frequency of the bamboo wood was 80 Hz; and that of the carbon fiber was 125 Hz. The intrinsic frequency of beetle wings was ~130 Hz, which was closer to the frequency of carbon wings, so carbon fiber was chosen as the material for the artificial wing veins.

The wing cable-driven folding mechanism was designed to achieve the PASFD function. Spanwise folding and deployment required the hinge joints to achieve rotation, and elastic hinges instead of normal hinge joints have been shown to be well suited for micro-robots [38]. We chose nitrile rubber (elasticity modulus: 6.1 MPa, Poisson’s ratio: 0.49) as the fabrication material for the elastic hinge. Then, the cable-driven method was chosen to drive the elastic hinge. As shown in Figure 3c, the end of the nylon cord OB was glued at the 1/3 position of the folded section. When the nylon cord was pulled, the wing-tip rotated around hinge A to reach the AB′ position. Since ∠OAB approached a flat angle, it was approximated as a straight line. When the folding angle △F was designed to be 60° and the length of OB was given, the length variation of the nylon cable was calculated by the cosine theorem as △L = L − L′ ≈ 2.6 mm. Figure 3d demonstrates the relationship between the phase angle of the cam, the length of the nylon cord, and the folding angle of the wing.

### 3.5. Simulation Setup

The simulation setup, calculation, and postprocessing for kinematic verification of the RURS-based flapping-wing drive mechanism were carried out with ADAMS software (ADAMS 2019, Mechanical Dynamics Inc., CA, USA). A slightly simplified model of the RURS mechanism, with the necessary mechanism rods and without the gear mechanism and mounting base, was used for the simulation. After the moving pair constraints were configured, its driving speed was set to 1440 rad/s (corresponding to a flapping frequency of 40 Hz). A marker point attached to the end of the output rod was set to track the “8” shape trajectory of the RURS mechanism.

## 4. Conclusions and Future Work

Herein, we observed and reconstructed the stroke motion trajectories of flying beetles, and found the wing-tip-folding phenomena under visual stimulation with circular optical flow. The flight forces data measured by the force sensor reveal the functions of wing-tip folding on the aerodynamics of flying beetles. The tethered flight experiments showed that the PASFD of the wing-tips of beetles significantly alters aerodynamic forces without any changes in flapping frequency. Inspired by this, a micro-flapping-wing mechanism prototype with PASFD was designed, fabricated, and tested in the motion capture and force measurement system to validate its kinematic functions and aerodynamic performance under different operating conditions. The flapping angles and folding angle testing results indicate that the proposed beetle-inspired flapping mechanism fits well with the stroke motion angles’ variation range of flying beetles. Aerodynamic forces of the prototype were evaluated to demonstrate aerodynamic performance. The aerodynamic experimental results imply that a larger flapping frequency results in a higher flight force, but the increasing trend is different under various operating conditions. Specifically, unilateral folding and deployment of the wings produced significant lateral thrust (7.65 mN with left-wing folding, and 8.52 mN with right-wing folding). Moreover, by altering the DC motor supply voltage, we were able to flexibly change the aerodynamic forces of the flapping-wing mechanism prototype, offering potential for applications such as flapping-wing micro-air vehicles with rapid ascent and high maneuverability.

This article is a foundational step towards a miniature, fully autonomous FWMAV. In the near future, we would like to develop a fine aerodynamic model of a miniature flapping-wing mechanism to further analyze the flow field distribution and aerodynamic characteristics of artificial wings. Meanwhile, optimized design of the flapping-wing mechanism is also necessary to obtain a more reliable structure that sustains higher flapping frequencies, resulting in a wider range of aerodynamic adjustments. The future of FWMAVs looks promising as research continues to improve their maneuverability, stability, and payload capacity. With further advancements in materials science and manufacturing techniques, it may be possible to create even more agile MAVs that can replicate the flight capabilities of insects even more closely. As we continue to push the boundaries of FWMAV technology, we can expect to see a growing impact on various fields where aerial vehicles are needed for practical applications.

## Figures and Tables

**Table 1:** Material properties of carbon fiber, polyformaldehyde resin, and bamboo wood.

Material Type	Density (g/cm^3^)	Elastic Modulus (GPa)
Carbon fiber	1.4~1.8	120~240
Polyformaldehyde resin	1.42	2~4
Bamboo wood	0.6~0.8	20~30

## Data Availability

The data are included in this article.

## References

[1] Bi Y., Lan M., Li J., Lai S., Chen B.M. (2019). A lightweight autonomous MAV for indoor search and rescue. Asian J. Control.

[2] Lindqvist B., Kanellakis C., Mansouri S.S., Agha-Mohammadi A.-A., Nikolakopoulos G. (2022). COMPRA: A COMPact Reactive Autonomy Framework for Subterranean MAV Based Search-And-Rescue Operations. J. Intell. Robot. Syst..

[3] Solis J., Karlsson C., Johansson S., Richardsson K. (2021). Towards the Development of an Automatic UAV-Based Indoor Environmental Monitoring System: Distributed Off-Board Control System for a Micro Aerial Vehicle. Appl. Sci..

[4] Hou K., Tan T., Wang Z., Wang B., Yan Z. (2023). Scarab Beetle-Inspired Embodied-Energy Membranous-Wing Robot with Flapping-Collision Piezo-Mechanoreception and Mobile Environmental Monitoring. Adv. Funct. Mater..

[5] Zhang J., Zhao N., Qu F. (2023). Bio-inspired flapping wing robots with foldable or deformable wings: A review. Bioinspir. Biomim..

[6] Ma K.Y., Chirarattananon P., Fuller S.B., Wood R.J. (2013). Controlled Flight of a Biologically Inspired, Insect-Scale Robot. Science.

[7] Chen Y., Wang H., Helbling E.F., Jafferis N.T., Zufferey R., Ong A., Ma K., Gravish N., Chirarattananon P., Kovac M. (2017). A biologically inspired, flapping-wing, hybrid aerial-aquatic microrobot. Sci. Robot..

[8] Graule M.A., Chirarattananon P., Fuller S.B., Jafferis N.T., Ma K.Y., Spenko M., Kornbluh R., Wood R.J. (2016). Perching and takeoff of a robotic insect on overhangs using switchable electrostatic adhesion. Science.

[9] Chen Y., Zhao H., Mao J., Chirarattananon P., Helbling E.F., Hyun N.-S.P., Clarke D.R., Wood R.J. (2019). Controlled flight of a microrobot powered by soft artificial muscles. Nature.

[10] James J., Fuller S. (2021). A high-voltage power electronics unit for flying insect robots that can modulate wing thrust. Proceedings of the 2021 IEEE International Conference on Robotics and Automation (ICRA).

[11] Hines L., Campolo D., Sitti M. (2014). Liftoff of a Motor-Driven, Flapping-Wing Microaerial Vehicle Capable of Resonance. IEEE Trans. Robot..

[12] Zou Y., Zhang W., Zhang Z. (2016). Liftoff of an Electromagnetically Driven Insect-Inspired Flapping-Wing Robot. IEEE Trans. Robot..

[13] Lau G.-K., Lim H.-T., Teo J.-Y., Chin Y.-W. (2014). Lightweight mechanical amplifiers for rolled dielectric elastomer actuators and their integration with bio-inspired wing flappers. Smart Mater. Struct..

[14] Phan H.V., Kang T., Park H.C. (2017). Design and stable flight of a 21 g insect-like tailless flapping wing micro air vehicle with angular rates feedback control. Bioinspir. Biomim..

[15] Gong D., Lee D., Shin S., Kim S. (2019). String-based flapping mechanism and modularized trailing edge control system for insect-type FWMAV. Int. J. Micro Air Veh..

[16] Phan H.V., Aurecianus S., Kang T., Park H.C. (2019). KUBeetle-S: An insect-like, tailless, hover-capable robot that can fly with a low-torque control mechanism. Int. J. Micro Air Veh..

[17] Phan H.V., Park H.C. (2020). Mechanisms of collision recovery in flying beetles and flapping-wing robots. Science.

[18] Karásek M., Muijres F.T., De Wagter C., Remes B.D.W., de Croon G.C.H.E. (2018). A tailless aerial robotic flapper reveals that flies use torque coupling in rapid banked turns. Science.

[19] Tijmons S., Karásek M., de Croon G. (2018). Attitude control system for a lightweight flapping wing MAV. Bioinspir. Biomim..

[20] Elzinga M.J., van Breugel F., Dickinson M.H. (2014). Strategies for the stabilization of longitudinal forward flapping flight revealed using a dynamically-scaled robotic fly. Bioinspir. Biomim..

[21] Beatus T., Guckenheimer J.M., Cohen I. (2015). Controlling roll perturbations in fruit flies. J. R. Soc. Interface.

[22] Sun M. (2014). Insect flight dynamics: Stability and control. Rev. Mod. Phys..

[23] Wootton R. (2000). From insects to microvehicles. Nature.

[24] Li Y., Cao F., Doan T.T.V., Sato H. (2016). Controlled banked turns in coleopteran flight measured by a miniature wireless inertial measurement unit. Bioinspir. Biomim..

[25] Haas F., Gorb S., Blickhan R. (2000). The function of resilin in beetle wings. Proc. R. Soc. B Biol. Sci..

[26] Chapman R.F. (2013). The Insects: Structure and Function.

[27] Fu F., Li Y., Wang H., Li B., Sato H. (2022). The function of pitching in Beetle’s flight revealed by insect-wearable backpack. Biosens. Bioelectron..

[28] Ajanic E., Feroskhan M., Mintchev S., Noca F., Floreano D. (2020). Bioinspired wing and tail morphing extends drone flight capabilities. Sci. Robot..

[29] Cong M., Li J. (2019). Design and analysis of three-dimensional bio-inspired flapping-wing mechanism based on spatial RURS linkage. J. Aerosp. Power.

[30] Vance J.T., Altshuler D.L., Dickson W.B., Dickinson M.H., Roberts S.P. (2014). Hovering Flight in the Honeybee *Apis mellifera*: Kinematic Mechanisms for Varying Aerodynamic Forces. Physiol. Biochem. Zool..

[31] Combes S.A., Gagliardi S.F., Switzer C.M., Dillon M.E. (2020). Kinematic flexibility allows bumblebees to increase energetic efficiency when carrying heavy loads. Sci. Adv..

[32] Gau J., Gemilere R., Lynch J., Gravish N., Sponberg S., LDS-VIP (FM Subteam) (2021). Rapid frequency modulation in a resonant system: Aerial perturbation recovery in hawkmoths. Proc. R. Soc. B Biol. Sci..

[33] Li Y., Sato H., Li B. (2021). Feedback Altitude Control of a Flying Insect–Computer Hybrid Robot. IEEE Trans. Robot..

[34] Li Y., Wu J., Sato H. (2018). Feedback Control-Based Navigation of a Flying Insect-Machine Hybrid Robot. Soft Robot..

[35] Oh S., Lee B., Park H., Choi H., Kim S.-T. (2020). A numerical and theoretical study of the aerodynamic performance of a hovering rhinoceros beetle (*Trypoxylus dichotomus*). J. Fluid Mech..

[36] Nedunchezian K. (2019). Effects of Flapping Wing Kinematics on the Aeroacoustics of Hovering Flight. J. Sound Vib..

[37] Naka H., Hashimoto H. (2015). Effects of deformation and vibration characteristics of wings on flapping flight. Mech. Eng. J..

[38] Zheng W., Zhao F. (2003). Research on flexible hinges. Opt. Precis. Eng..

